# Prevalence and Associated Risk Factors of Sarcopenia Among Elderly With Diabetes in Japan and Taiwan

**DOI:** 10.1093/geroni/igaa057.574

**Published:** 2020-12-16

**Authors:** Yuko Yamaguchi, Chieko Greiner, Hirochika Ryuno, Pi-Hsia Lee, Hsin-Yen Yen, Shu-Chun Lee, Chiou-Fen Lin, Ting-I Lee

**Affiliations:** 1 Kobe University, Kobe, Japan; 2 KOBE UNIVERSITY, Kobe, Japan; 3 Taipei Medical University, Taipei, Taiwan (Republic of China)

## Abstract

The aim of this study was to investigate the prevalence of sarcopenia and its associated risk factors among elderly with diabetes in Japan and Taiwan. A cross-sectional study was conducted through convenience sampling. This study was approved by the institutional review boards of Kobe University Graduate School of Health Sciences (No. 543) and Taipei Medical University (No. N201905065). Of the 114 Japanese participants (24.9% females) and 226 Taiwanese participants (75.1% females), the mean age were 73.7±6.9 years and 74.2±6.6 years, respectively. Sarcopenia was found in 10.6% in Japan and 11.1% in Taiwan with no significant difference between both countries. Older age, poor relationships with neighbors, and poor consciousness for health management were significantly associated with sarcopenia in both countries. Additionally, Japanese participants with sarcopenia had significantly longer duration of diabetes, higher level of emotional distress assessed by the Problem Areas In Diabetes (PAID) scale, and lower level of total protein, but no significant differences were shown in Taiwanese participants. Binomial logistic regression analyses were performed to detect risk factors related to sarcopenia. After adjusting for age, sarcopenia was found to be significantly associated with poor consciousness for health management in Japanese participants (OR:0.12 P=0.02) and significantly associated with poor relationships with neighbors and poor consciousness for health management in Taiwanese participants (OR:0.18 P=0.008 and OR:0.45 P=0.04, respectively). These findings are expected to contribute to the development of effective strategies for preventing sarcopenia among elderly with diabetes in Japan and Taiwan.

